# Neuropilin‐1 is up‐regulated by cancer‐associated fibroblast‐secreted IL‐8 and associated with cell proliferation of gallbladder cancer

**DOI:** 10.1111/jcmm.15825

**Published:** 2020-09-20

**Authors:** Chen Chen, Rui Zhang, Li Ma, Qi Li, Ya‐ling Zhao, Guan‐jun Zhang, Dong Zhang, Wen‐zhi Li, Sheng Cao, Lin Wang, Zhi‐min Geng

**Affiliations:** ^1^ Department of Hepatobiliary Surgery The First Affiliated Hospital of Xi’an Jiaotong University Xi’an Shaanxi Province China; ^2^ Department of Emergency The First Affiliated Hospital of Xi'an Jiaotong University Xi’an Shaanxi Province China; ^3^ Department of of Epidemiology and Biostatistics School of Public Health Xi’an Jiaotong University Health Science Center Xi’an Shaanxi Province China; ^4^ Department of Pathology The First Affiliated Hospital of Xi'an Jiaotong University Xi’an Shaanxi Province China; ^5^ Gastroenterology Research Unit Division of Gastroenterology and Hepatology Mayo Clinic Rochester MN USA

**Keywords:** cancer‐associated fibroblasts, cell proliferation, gallbladder cancer, neuropilin‐1, prognostic factor

## Abstract

We previously demonstrated that cancer‐associated fibroblasts (CAFs) promoted the proliferation of gallbladder cancer (GBC) cells, but the mechanism is not clear. Neuropilin‐1 (*NRP‐1*) plays an important role in various malignancies as transmembrane glycoprotein. Our goal was to reveal the relationship between CAFs and *NRP‐1* and their potential functions in GBC. In this study, we found *NRP‐1* was overexpressed in GBC tissue, associated with poor survival and was up‐regulated by CAFs. The cytokine array cluster analysis revealed IL‐8 secreted by CAFs facilitated the up‐regulation of *NRP‐1* in tumour cells. *NRP‐1* knockdown suppressed tumour growth in vivo. Gene expression microarray analysis showed 581 differentially regulated genes under *NRP‐1* knockdown conditions. Ingenuity pathway analysis demonstrated that *NRP‐1* knockdown may inhibit tumour progression by affecting cell proliferation. We then confirmed that *NRP‐1* knockdown in NOZ and GBC‐SD cells significantly inhibited cell proliferation. Additionally, the IL‐8 mediated MDM2 and CCNA2 expression were affected by *NRP‐1* knockdown. Our findings suggested that *NRP‐1* was up‐regulated by CAF‐secreted IL‐8, which subsequently promoted GBC cell proliferation, and these molecules may serve as useful prognostic biomarkers and therapeutic targets for GBC.

**Abbreviations**
CAFs: cancer‐associated fibroblasts
GBC: gallbladder cancer
IPA: Ingenuity Pathway Analysis
NFs: normal fibroblasts
NRP‐1: Neuropilin‐1
sh RNA: short hairpin RNA
TMA: tissue microarray

## 1 INTRODUCTION

Gallbladder cancer (GBC) is the most common malignant tumour of the biliary system.^1^ GBC is associated with poor treatment response and prognosis because it is difficult to diagnose early and has a high propensity to metastasize to lymph nodes.^2^

Biliary malignancies are characterized by interstitial fibrosis,^3^ and cancer‐associated fibroblasts (CAFs) have been shown to be directly associated with patient outcomes in a variety of tumours, including cholangiocarcinoma.^4^ Our previous study demonstrated that CAFs promoted the proliferation and invasion of GBC cells.^5^ However, which cytokines are secreted by CAFs and how these cytokines act on GBC cells remain unclear.

Neuropilin‐1 (*NRP‐1*) was involved in the progress of many cancers,^6^ it is found overexpressed in hepatocellular carcinoma,^7^ colorectal cancer,^8^ glioblastoma,^9^ and lung cancer,^10^ and its expression associated with advanced cancer stages and poor prognosis.^11^, ^12^, ^13^, ^14^

The mechanisms for the role of *NRP‐1* in cancer progression rely on its interactions with several key signalling pathways in cancer cells, such as transforming growth factor β1 (TGFβ1), semaphorin (Sema), vascular endothelial growth factor (VEGF), hedgehog (HH), interferon‐γ (IFNγ) and GAIP/RGS19‐interacting protein (GIPC1).^15^, ^16^, ^17^, ^18^, ^19^, ^20^ Therefore, targeting *NRP‐1* has been shown to be a potential therapeutic target for some cancers.^21^, ^22^ However, the role of *NRP‐1* in GBC remains unclear. This study investigated the interactions between CAFs and *NRP‐1* and clarified their biological and prognostic role in GBC tumorigenesis.

## 2 MATERIALS AND METHODS

### 2.1 Patient tissue samples and follow‐up

A total of 91 patients (The First Affiliated Hospital of Xi'an Jiaotong University, China, 2008‐2013) with pathologically confirmed GBC were included in this cohort. The diagnosis and pathological evaluation were according to the World Health Organization defining criteria (2010).^23^ The TNM staging was according to the 7th AJCC criteria.^24^ The study was approved by the Ethics Committee of the First Affiliated Hospital of Xi'an Jiaotong University, China.

The data cut‐off point of follow‐up was August 2016, and overall survival (OS) was defined as the time interval from surgery to death.

### 2.2 Tissue microarray, immunohistochemistry and immunofluorescence

Tumour microarray (TMA) was constructed using paraffin‐embedded GBC tissue and cholecystitis. We performed immunohistochemistry using a two‐step method.

The TMA sections were incubated with first antibody overnight at 4°C and then incubated with secondary antibodies for 1 hour. The cell nuclei were counterstained with haematoxylin (H8070, Solarbio Beijing, China) for 30 seconds. Diaminobenzidine (DA1010, Solarbio, Beijing, China) was used for visualization of positive staining. We scored positive cell staining as follows: 0 point: <10% positive cells, 1 point: 11%‐50%, 2 points: 51%‐75% and 3 points: >76%. We classified cytoplasmic staining as follows: 0 points: no staining, 1 point: yellow, 2 points: brown and 3 points: tan and the protein expression was categorized by the sum of these two scores: negative (−): 0‐1 point, positive (+, low expression): 2‐3 points and strong positive (++, high expression): 4‐6 points.

Immunofluorescence analysis of IL‐8 was performed with NFs and CAFs. The cells were fixed with 4% paraformaldehyde for 10 minutes, permeabilized with 0.1% Triton™ X‐100 for 10 minutes and blocked with 1% BSA for 1 hour at room temperature. The cells were labelled with IL‐8 antibody and incubated overnight at 4°C and then labelled with Goat Anti‐Mouse IgG H&L for 1 hour at room temperature (Panel A: green). Nuclei (Panel B: blue) were stained with DAPI. The images were captured at 100× magnification.

### 2.3 Cell, antibodies, and reagents

We purchased GBC‐SD, NOZ and SGC‐996 cell lines from Chinese Academy of Sciences Cell Bank (Shanghai, China) and cultured GBC cell lines with RPMI‐1640. All cells used in this study were authenticated by short tandem repeat DNA profiling by Genetica, and mycoplasma contamination was routinely tested using detection kit (CA1080, Solarbio).

We isolated CAFs and normal fibroblasts (NFs) from 4 different GBC patients (all these 4 patients with T3N0M0 adenocarcinoma) and 4 different chronic cholecystitis patients (all these 4 patients were young men less than 35 years old and with a history of gallstone less than one year), using previously described digestion methods.^5^

These antibodies and reagents were used in this study: anti‐*NRP‐1* (Rabbit polyclonal, ab25998, Abcam), anti‐Murine double minute 2 (anti‐MDM2; Rabbit polyclonal, ab38618, Abcam), anti‐Cyclin A2 (anti‐CCNA2; Mouse monoclonal, BF683, Cell Signaling), anti‐Early Growth Response 1 (anti‐EGR1; Rabbit monoclonal, 44D5, Cell Signaling), anti‐IGF1R (Rabbit polyclonal, ab39675, Abcam), anti‐GAPDH (Mouse Monoclonal, AM4300, Invitrogen), anti‐IL‐8 (Mouse monoclonal, M801, Thermo), secondary antibody to Mouse IgG‐H&L (Goat polyclonal, ab150113, Abcam), IL‐8 receptors inhibitor Reparixin (HY‐15251, MCE) and Human IL‐8 ELISA Kit (KE00006, Proteintech Group).

### 2.4 Determination of cytokines secreted by CAFs

Four cases of CAFs and four cases of NFs were plated in 10mm dishes (3*10^4^ cells each dish) and cultured for 72 hours, and cell supernatants were collected. Following the standard operating procedures for Human Cytokine Array G5 (AAH‐CYT‐G5) protein array kit (RayBiotech), 20 µL supernatant from each tube was used to assess the difference of cytokines expression between CAFs and NFs. Later, we obtained the expression levels by using fluorescence scanning (GenePix 4000B, Axon Instruments, Inc) and analysis with a built‐in data transformation tool. After that, we confirmed IL‐8 was highly expressed in the CAFs supernatant by ELISA. Furthermore, to investigate whether NRP‐1 was up‐regulated by the CAFs‐secreted IL‐8, GBC cells were divided into three groups: GBC cells as control group; cells treated with CAFs supernatants for 48 hours; cells treated with CAFs supernatants combined Reparixin (0.1 μmol/L) for 48 hours; and the expression of NRP‐1 were tested.

### 2.5 Construction of lentiviral vectors and cell transfection

The target sequence for *NRP‐1* shRNA was GAGAGAACAAGGTGTTCAT. The hU6‐MCS‐CMV‐RFP lentiviral plasmid was used as a vector. After polymerase chain reaction identification, we infected GBC‐SD and NOZ cells with positive lentiviral vectors (*NRP‐1* shRNA) in 1640 medium plus 10% foetal bovine serum with Eni. S + polybrene (3 × 10^8^ TU/mL), and we used 8 μL empty lentiviral vector (NT shRNA, 5 × 10^8^ TU/ml) as negative controls. Images of fluorescence intensity were captured 96 hours after infection.

### 2.6 **Tumo**u**r formation in nude mice**

All mice were housed and handled under the guidelines of Institutional Animal Care and Use Committee of Xi'an Jiaotong University. The sh‐*NRP‐1*‐GBC‐SD or NT sh‐Ctrl cells were digested and made into cell suspensions. After counting, the cells were kept at 4°C for further use. Twelve 4‐ to 6‐week‐old healthy nude mice (SJA Laboratory Animal Co., Ltd.) were allocated into two groups randomly. 1 × 10^6^ cells were subcutaneously injected to the lower limbs. Tumour size was measured every 3 days, and the mice were killed 3 weeks after injection.

### 2.7 Ingenuity Pathway Analysis

Ingenuity Pathway Analysis (IPA; Ingenuity H Systems, Redwood City, CA, USA; http://www.ingenuity.com) was used to analyse the gene chips data. The quality of these analyses was confirmed by Signal Histogram, Pearson's correction and Relative Signal Box Plot. The Ingenuity Pathway Knowledge Base (IPKB), which derives from the known functions and published interactions of genes, is the basis of IPA, and it allows the predicting of function and pathways for a particular gene. Differentially expressed genes (DE genes) were located into the Ingenuity database genetic networks and then ranked by score. In our study, we set significance levels at fold change >|2|, analysis of variance (ANOVA)*P* < .05 and false discovery rate (FDR) *q* < 0.05 for identifying DE genes.

### 2.8 Cellular proliferation assay

We determined cell proliferation rates using MTT assay. GBC cells infected with *NRP‐1* shRNA or NT shRNA were seeded in 96‐well plates (2 × 10^3^ cells per well), after incubated at 37°C for 24 and 48 hours, MTT solution was added and incubated for 4 hours, and then, the absorbance values at 490 nm were obtained. For the cell colony formation assays, we seeded 1000 cells of each group per well in a 60 mm dish and incubated for 14 days, fixed the cells with 4% paraformaldehyde and stained with crystal violet, and those colonies with more than 50 cells were count.

### 2.9 Western blot analysis

RIPA buffer (100‐150 µL) containing NaF, Na_2_VO_3_ and PMSF was added to the cell dishes and incubated for 30 minutes on ice, the cells were scraped and the lysates were collected and cleared by centrifugation. DC™ Protein Assay kit (Bio‐Rad Laboratories) was used to quantify the protein concentrations. Appropriate amounts of total protein were loaded into each well of a sodium dodecyl sulphate gel for electrophoresis, followed by standard blotting and antibody incubation procedures. Antibody dilutions used for WB were as follows: anti‐*NRP‐1* (rabbit, 1:1000), anti‐MDM2 (mouse polyclonal, 1:3000), anti‐CCNA2 (1:500), anti‐IGF‐1R (rabbit, 1:1000), anti‐EGR1 (1:1000) and anti‐GAPDH (1:5000).

### 2.10 Statistical analysis

We performed statistical analysis using SPSS 14.0 (SPSS Inc IBM). Independent‐sample *t* test and ANOVA test was used for comparisons between two groups and multiple groups, respectively. Chi‐square test of independence was used to determine whether there is a significant difference between categorical variables. Survival was analysed using the Kaplan‐Meier method, and the differences were measured by log‐rank tests. Cox regression was carried out for prognostic multivariate analysis. A *P* < .05 was defined as statistically significant.

## 3 RESULTS

### 3.1 *NRP‐1* expression in GBC tissues and cholecystitis tissues

**FIGURE 1:**
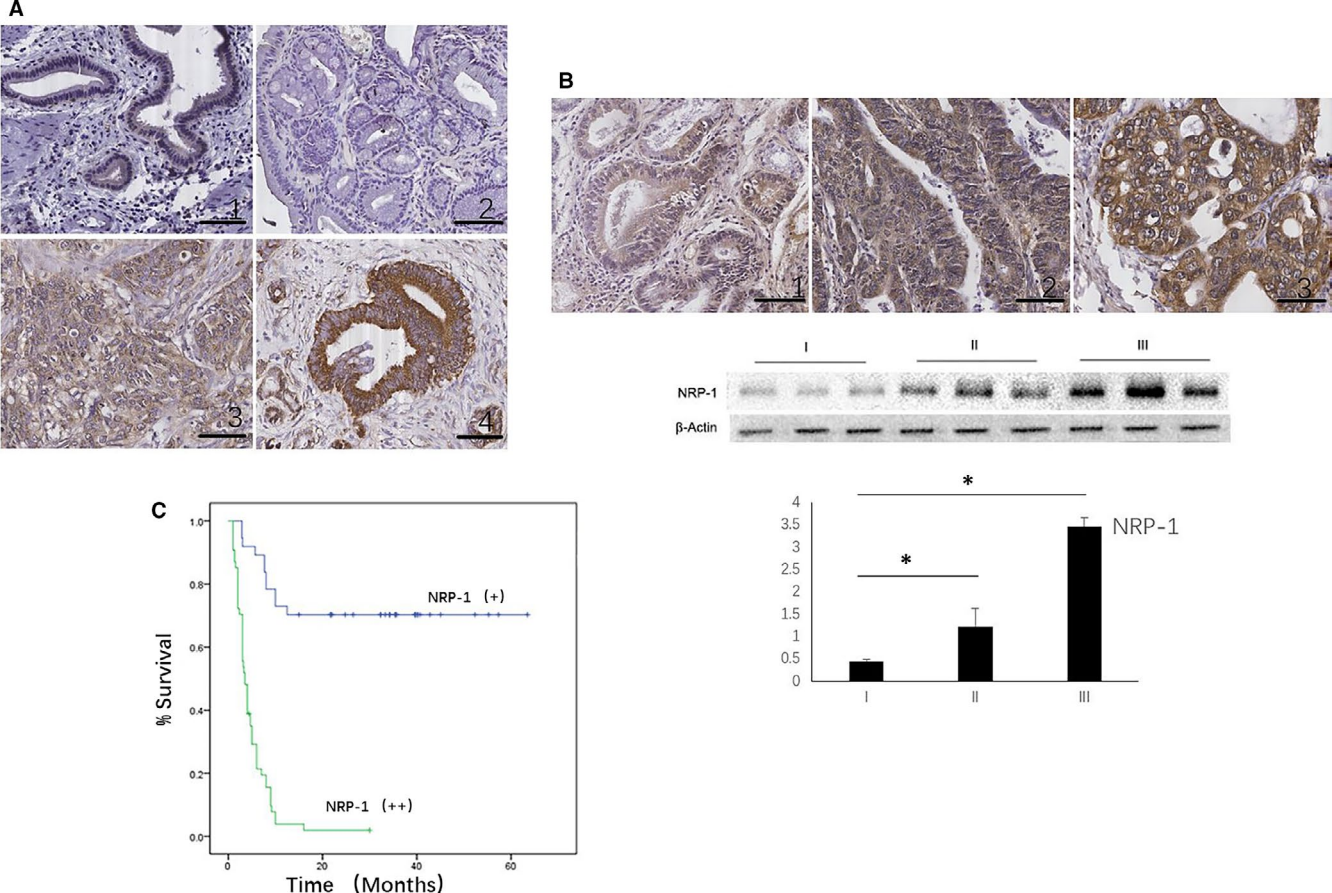
Expression and clinical significance of NRP‐1 in gallbladder cancer (GBC). (A) NRP‐1 expression in different tissues. 1.NRP‐1 expression in chronic cholecystitis tissue; 2.NRP‐1 expression in acute cholecystitis tissue; 3‐4. NRP‐1 expression in GBC tissue. (200×) (B) The expression of NRP‐1 in GBC tissue samples with different pathological differentiation. 1. Grade I adenocarcinoma. 2. Grade II adenocarcinoma. 3. Grade III adenocarcinoma. Patients with high NRP‐1 expression had worse pathological differentiation, *P < .05 by ANOVA. (C) Overall survival (OS) curve for a cohort of GBC patients with different NRP‐1 expression. Patients with high NRP‐1 expression had worse OS (*P* < .01)

We examined *NRP‐1* expression in 91 GBC samples and in 120 cholecystitis tissues (60 acute cholecystitis and 60 chronic cholecystitis) using immunohistochemistry (IHC). The *NRP‐1* protein expression positive rate was 100% in GBC tumours (40.7% positive and 59.3% strong positive), 36.7% in acute cholecystitis (63.3% negative, 30% positive and 6.7% strong positive) and 58.3% in chronic cholecystitis (41.7% negative, 48.3% positive and 10% strong positive) (*P* < .01, χ^2^ = 99.6), indicating the expression of *NRP‐1* was significantly increased in GBC tumours (Figure 1A). In GBC, comparing with patients who had low *NRP‐1* expression, patients with high *NRP‐1* expression had worse TNM staging (*P* < .01) and pathological differentiation (*P* < .01) (Figure 1B), and the expression of *NRP‐1* also consistent with the CAFs activation marker α‐SMA (Table 1).

**TABLE 1:** Correlation between NRP‐1 expression and GBC clinicopathological characteristics

Characteristics	NRP‐1			*P‐*value	Correlation coefficient
	Total	Low	High		
Pathology differentiation					
I	15	15	0	<.01	0.529
II	39	15	24		
III	37	7	30		
TNM staging					
I‐II	7	7	0	<.01	0.615
III	33	24	9		
IV	51	6	45		
α‐SMA expression					
Low	23	20	3	<.01	0.548
High	68	17	51		
Sex					
Male	34	13	21	.83	
Female	57	24	33		
Age					
<60	29	12	17	1.00	
≥60	62	25	37		

### 3.2 High *NRP‐1* expression associated with poor survival

Kaplan‐Meier survival analysis of patients with different expression levels of *NRP‐1* showed that the median survival time (MST) was 33 months in the low *NRP‐1* expression group, in contrast, MST of the high *NRP‐1* expression group was only 4 months (*P* < .01, Figure 1C). Multivariate analysis showed that surgical margin, *NRP‐1* expression and pathological differentiation were all independent risk factors for poor prognosis (Table 2).

**TABLE 2:** Multivariate analysis for prognostic factors in GBC patients

Variables	OR (95% CI)	*P* value
Sex		
Male	1.00 (reference)	
Female	1.933 (0.87‐4.30)	.105
Age		
<60	1.00 (reference)	
≥60	0.506 (0.283‐0.904)	.506
NRP1 expression		
(+)	1.00 (reference)	
(++)	4.867 (2.00‐11.86)	<.01
Surgery		
R0 resection	1.00 (reference)	
R1/2 resection	2.42 (1.291‐4.543)	<.01
TNM staging		
Stage IV	1.00 (reference)	
Stage III	0.256 (0.09‐0.723)	<.01
Stage I‐II	0.000 (0.00‐0.00)	.970
Pathology differentiation		
Grade III	1.00 (reference)	
Grade II	0.241 (0.14‐0.42)	<.01
Grade I	0.570 (0.14‐2.26)	.423

### 3.3 CAFs‐secreted IL‐8 up‐regulated *NRP‐1*

**FIGURE 2:**
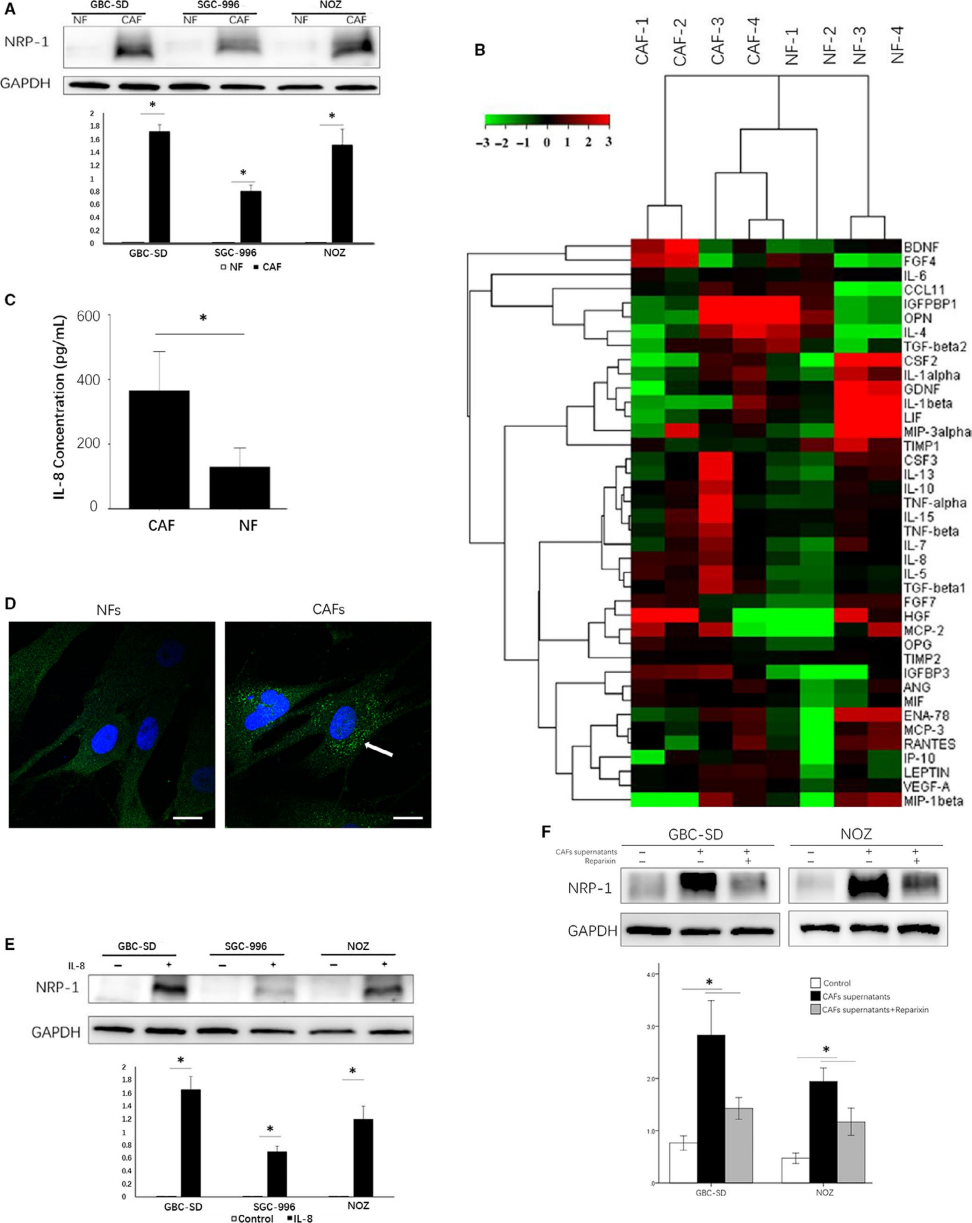
CAFs up‐regulated NRP‐1 expression by IL‐8 in gallbladder cancer (GBC). (A) CAFs up‐regulated NRP‐1 expression in different GBC cell lines (GBC‐SD, SGC‐996 and NOZ). (B) Cytokine array cluster analysis of the exocrine cytokines in CAFs and NFs. (C) The IL‐8 expression in CAFs and NFs were determined by ELISA Kit. (D) IL‐8 in NFs and CAFs cells. Green: IL‐8 (marked with arrow); Blue: nuclei (stained with DAPI). Scale bar: 25 μm. (E) IL‐8 up‐regulated NRP‐1 expression in different GBC cell lines (GBC‐SD, SGC‐996 and NOZ). (F) IL‐8 receptors inhibitor Reparixin reduced the expression of NRP‐1 in GBC cell lines which up‐regulated by CAFs supernatants (GBC‐SD, NOZ). *P < .05 by ANOVA

We treated GBC cell lines (GBC‐996, GBC‐SD and NOZ) with conditioned supernatants from CAFs and NFs, and we found that CAFs supernatant up‐regulated *NRP‐1* expression level in GBC cell lines (Figure 2A). We used cytokine array cluster analysis to compare the exocrine cytokines of CAFs and NFs, and we found that IL‐8 and IGFBP3 secreted by CAFs were higher than that by NFs (*P* < .05) (Figure 2B and Data S1). Furthermore, we confirmed that CAFs supernatants contained more IL‐8 than NFs supernatants by ELISA (365.7 pg/mL vs 129.3 pg/mL, *P* < .05) (Figure 2C), and we also performed the IL‐8 immunofluorescence in NFs and CAFs, which confirmed that CAFs secreted more IL‐8 than NFs (Figure 2D).

We next investigated whether NRP‐1 was up‐regulated by the CAFs‐secreted IL‐8, GBC cell lines were then treated with IL‐8 (100 μmol/L) and IGFBP3 (75 μmol/L). We found that IL‐8 up‐regulated the expression of *NRP‐1* (Figure 2E), whereas IGFBP3 had no significant effect on the expression of *NRP‐1*. Then, GBC‐SD and NOZ cells were treated with CAFs supernatants and Reparixin (0.1 μmol/L) for 48 hours, and we found that Reparixin significantly reduced the expression of NRP‐1 which should be up‐regulated by CAFs supernatants (Figure 2F). These results indicated that CAFs up‐regulation of *NRP‐1* in tumour cells was facilitated by secretion of IL‐8.

### 3.4 shRNA‐mediated inhibition of *NRP‐1* in GBC cells

**FIGURE 3:**
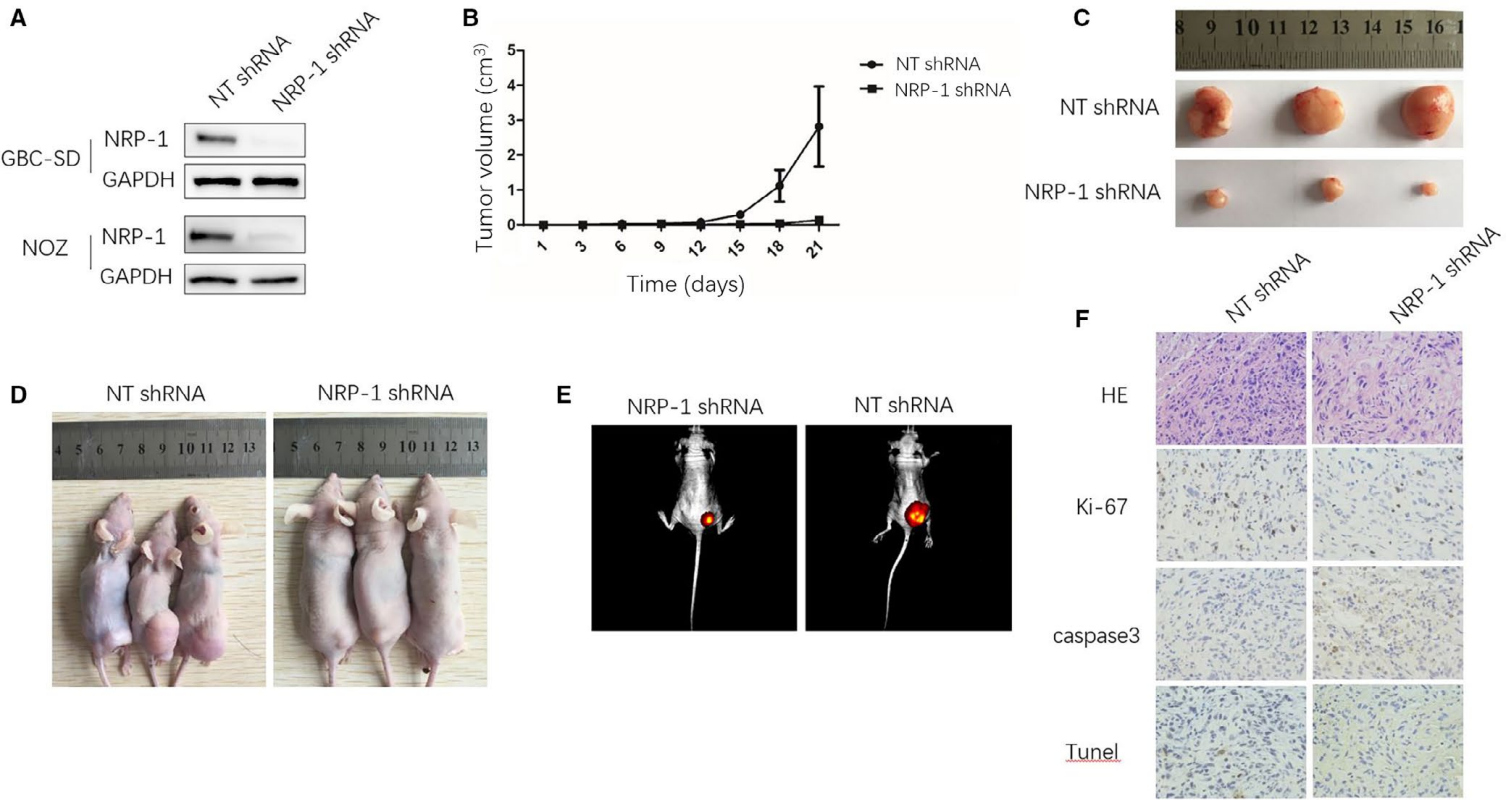
NRP‐1 knockdown impairs effects of GBC‐SD on tumour growth in mice. (A) Analysis of lentivirus mediated NRP‐1 shRNA knockdown, by using WB, we confirmed that the expression of NRP‐1 was knockdown both in GBC‐SD and NOZ cells. (B‐D) 0.5 × 10^6^ NT or NRP‐1 shRNA expressing GBC‐SD cells were implanted into nude mice by subcutaneous injection. Tumour size was measured each 3 days by a calliper. NRP‐1 knockdown GBC‐SDs were less effective on promoting tumour growth in mice. Tumour volume data were quantitated. (B) The tumour growth curve, (C)(D) the mice and tumours of different groups on the 21st day after injection, *P < .05 by ANOVA, n = 6 tumours. (E) GBC‐SD cells tagged with firefly luciferase were used for tumour co‐implantation. Bioluminescence of GBC‐SD was measured by a Xenogen IVIS 200 (Caliper Life Sciences). Representative mice are shown. (F) H&E staining, Ki67, caspase3 staining and TUNEL staining of mice tumours (100×), bar 50 μmol/L. In NRP‐1 shRNA tumour, TUNEL positive cells were significantly increased, the expression of Ki‐67 was decreased and Caspase‐3 was increased (*P* < .001)

*NRP‐1* shRNA was used to knockdown *NRP‐1* expression in GBC cell lines (GBC‐996, GBC‐SD and NOZ). Red fluorescent protein (RFP) detection revealed efficient lentiviral infection of GBC‐SD and NOZ cells, but inefficient infection of SGC‐996 cells. WB analysis revealed that *NRP‐1* protein expression dramatically decreased in GBC‐SD and NOZ cells after infection (Figure 3A). Cells in which *NRP‐1* was inhibited were named *NRP‐1* shRNA, and the native control cells were named NT shRNA.

### 3.5 **Suppression of *NRP‐1* attenuated tumo**u**r formation and growth**

Twelve mice were assigned to the *NRP‐1* shRNA and NT shRNA groups. We found that tumours grew more rapidly in the NT shRNA group as shown in Figure 3B. Mice were killed on the 21st day after injection, as Figure 3C–E shown, tumours from the NT shRNA group were much larger and heavier than tumours in the *NRP‐1* shRNA group (*P* < .01).

We performed TUNEL assay and IHC staining to evaluate proliferation and apoptosis. Comparing to the control group, in the *NRP‐1* shRNA group, the number of TUNEL positive cells was significantly increased and the expression of Ki‐67 in tumours was decreased (*P* < .001). We also found that Caspase‐3 protein expression was higher in the *NRP‐1* shRNA group than that in the control group (*P* < .001) (Figure 3F).

### 3.6 Gene chips analysis of *NRP‐1* inhibition

Gene chips were used on three samples each from the NT shRNA‐GBC‐SD and *NRP‐1* shRNA‐GBC‐SD, and pathway analysis was performed to explore the regulation and function of *NRP‐1* in GBC cells.

**FIGURE 4:**
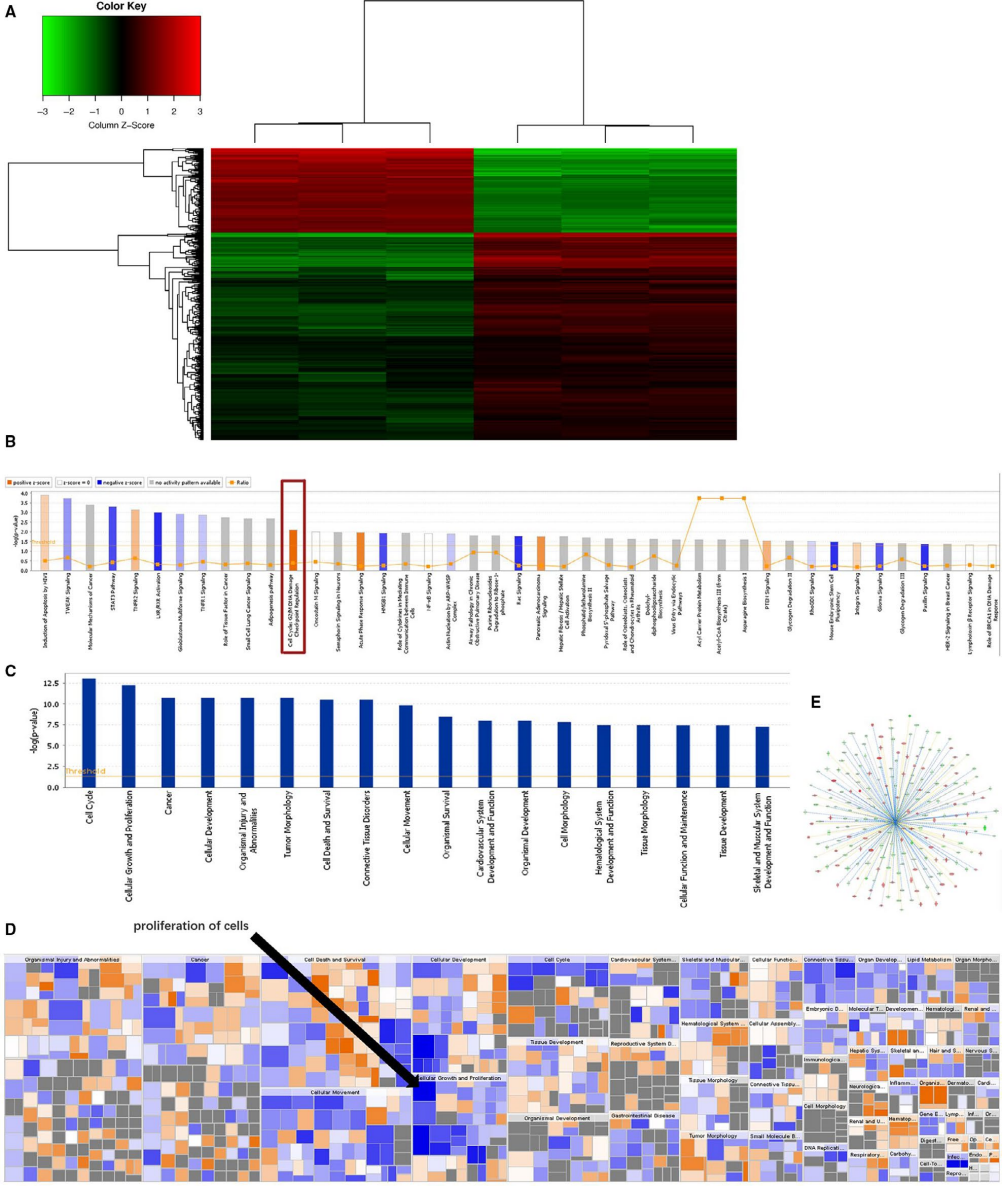
The heat map and diseases or bio functions analysis of NRP‐1 knockdown. (A) The up‐regulated genes were shown in red, and down‐regulated genes were shown in green, genes for which no obvious changes were observed are shown in black. (B) Enriched canonical pathways for differentially expressed genes in IPA. (C) Enrichment status of 18 categories of diseases or bio functions after NRP‐1 knockdown. (D) The heat map of diseases and bio functions of NRP‐1 knockdown. The square size was determined by –log (*P*‐value), while the colour was defined by the z‐score. The arrows illustrated the significantly enriched functional categories: proliferation of cells, which was decreased and ranked as the highest network. (E) The specific status of genes in proliferation of cells network, the up‐regulated genes were shown in red and down‐regulated genes were shown in green, the blue line indicates the expression status of the consistent inhibition between the upstream regulator and the gene, while the yellow line indicates the inconsistent expression status between the upstream regulator and the gene

A total of 48,726 publicly annotated gene clusters (Data S2) were identified in the microarray data, and 581 genes were differentially expressed among them (FC > |1.5|; *P* < .05) between the NT shRNA and *NRP‐1* shRNA groups (Data S3). Among them, 182 genes were up‐regulated and 399 genes were down‐regulated in the *NRP‐1* shRNA group. An interactive heat map has shown the abundance of these DE genes (Figure 4A). The top 20 down‐regulated and up‐regulated genes were listed in Tables S1 and S2.

### 3.7 Ingenuity Pathway Analysis of *NRP‐1* related biological functions

We next imported DE genes data sets into IPA to investigate the possible biological interactions and pathways. Among the 299 different canonical pathways identified by IPA, 43 were significantly enriched (*P* < .05), and 19 showed either positive or negative **z**‐scores (ie these pathways were activated or suppressed, respectively; see Data S4). Among these 19 pathways, *Cell Cycle: G2/M DNA Damage Checkpoint Regulation* was the only pathway with |**z**‐score | > 2, which meant it was significantly changed (Figure 4B).

IPA also grouped the DE genes into 18 functional categories, which included 500 diseases or bio functions (Data S5). The enrichment status of these 18 categories is presented in Figure 4C. The heat map showed the activated or suppressed state of these 500 diseases or bio functions after *NRP‐1* knockdown. Twenty‐one diseases or bio functions were significantly increased or decreased (|**z**‐score| > 2; Table S3). Among these significantly changed diseases and bio functions, *proliferation of cells* was decreased and ranked as the highest network with 206 focus molecules (z‐score = 4.357; Figure 4D), including *TGFBR1, EGR1, CCNA2, MDM2, BBC3, IGF2, RRas2* and *CDK6*. Figure 4E presents the specific status of genes in the *Proliferation of cells* network.

### 3.8 Suppression of *NRP‐1* inhibited growth and colony formation in GBC cells

**FIGURE 5:**
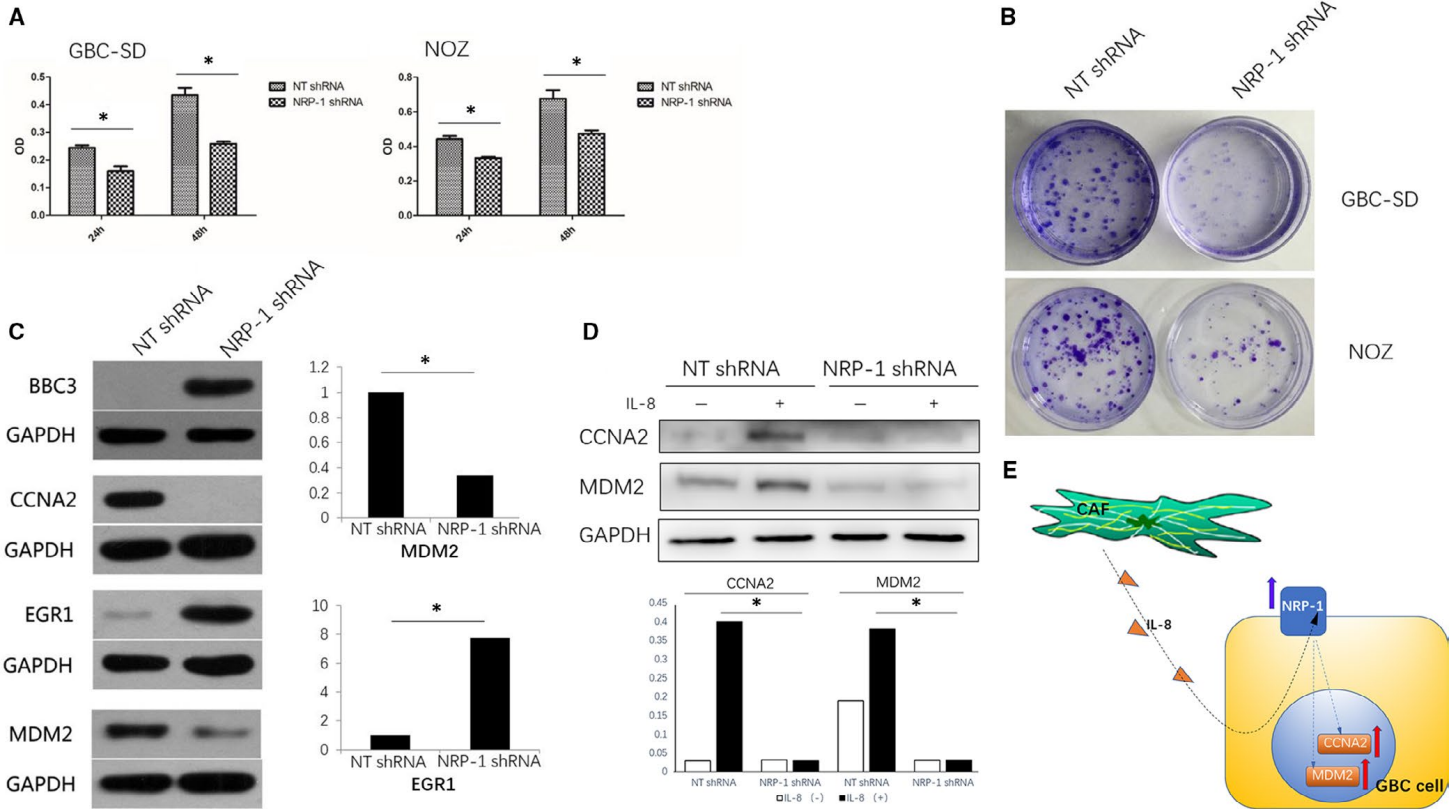
Effect of NRP‐1 knockdown on cell proliferation in GBC‐SD and NOZ cells. (A) MTT assay of NRP‐1 shRNA in GBC‐SD and NOZ cells, OD was decreased in NRP‐1 shRNA GBC‐SD and NOZ cells, *P < .05 by ANOVA. (B) Cell colonies assay of NRP‐1 shRNA in GBC‐SD and NOZ cells, the number of colonies was significantly lower in the NRP‐1 shRNA GBC‐SD and NOZ cells. (C) Western blot of the selected Gene Symbol, in NRP‐1 shRNA group, CCNA2, MDM2 protein expression was decreased and BBC3, EGR1 protein expression was increased, *P < .05 by ANOVA. (D) *NRP‐1* shRNA/ NT shRNA GBC‐SD cells were treated with IL‐8 (100 μmol/L), the up‐regulation of CCNA2 and MDM2 were blocked by *NRP‐1* shRNA, *P < .05 by ANOVA. (E) Schematic diagram of the mechanism by which CAFs secrete IL‐8 up‐regulated NRP‐1 in gallbladder cancer cells, subsequently promote GBC cell proliferation

IPA revealed that pathways and bio functions related to cell proliferation and cell cycle were significantly affected by *NRP‐1* knockdown. To confirm this finding, we conducted MTT assays. We found that cell proliferation was decreased in *NRP‐1* shRNA GBC‐SD and NOZ cells (Figure 5A). As the tumour colony formation assay showed (Figure 5B), in both GBC‐SD and NOZ cells, the number of colonies was significantly lower in the *NRP‐1* shRNA group (*P* < .01). This suggested that *NRP‐1* expression was associated with GBC cell proliferation.

We then analysed the expression of *NRP‐1*‐related genes at the protein level in *NRP‐1* shRNA (KD) and NT shRNA (NC) GBC‐SD cells. As shown in Figure 5C, CCNA2 protein expression was decreased and almost undetectable in the KD group, and MDM2 protein expression was also decreased by 66.3% in the KD group. In contrast, BBC3 protein was increased in the KD group. EGR1 protein expression was increased by 674% in the KD group. To further examine whether IL‐8 and *NRP‐1* affected these protein, *NRP‐1* shRNA/ NT shRNA GBC‐SD cells were treated with IL‐8 (100 μmol/L), and we found that *NRP‐1* shRNA blocked the up‐regulation of CCNA2 and MDM2(Figure 5D). Therefore, our results demonstrated that CAFs‐secreted IL‐8 up‐regulated *NRP‐1* in GBC cells and subsequently promoted GBC cell proliferation by decreasing the expression of CCNA2 and MDM2 (Figure 5E).

## 4 DISCUSSION

*NRP‐1* expression has been associated with prognosis in a variety of tumours, such as liver cancer, bladder cancer and colon cancer.^25^, ^26^ Although many studies have focused on *NRP‐1* function in tumour angiogenesis,^27^, ^28^, ^29^ only a few have examined the expression of *NRP‐1* on tumours cells.^30^ Additionally, there have been no published reports on *NRP‐1* function in GBC. The effect of CAFs on tumour cells is mainly achieved by paracrine cytokines, which play an extremely important role in promoting tumour proliferation, invasion and migration.^31^ Studies by Claperon^32^ have shown that HB‐EGF secreted by CAFs can promote angiogenesis and cell proliferation in cholangiocarcinoma. However, the functional role of CAFs in GBC remains unclear.

Our previous study demonstrated that CAFs promoted GBC cell proliferation.^5^ In this study, we found a clear link between *NRP‐1* expression and tumour differentiation, TNM staging and the CAFs activation marker α‐SMA. In that regard, higher *NRP‐1* expression was an independent risk factor for poor prognosis. Because CAFs have been associated with *NRP‐1* in GBC cells, we co‐cultured CAFs, NFs and GBC cell lines and found that CAFs up‐regulated *NRP‐1* in GBC cells. Next, by using a cytokine array and ELISA, we confirmed that CAFs secreted more IL‐8 than NFs. Therefore, we treated GBC cells with IL‐8 and showed that IL‐8 promoted *NRP‐1* expression, and the IL‐8 receptors inhibitor Reparixin reduced the expression of NRP‐1 which should be up‐regulated by CAFs supernatants. These results indicated that CAFs‐secreted IL‐8 up‐regulated *NRP‐1* in tumour cells.

A cancer cell xenograft model showed that *NRP‐1* knockdown inhibited tumour progression in vivo. To understand genome‐wide gene expression changes, we then performed mRNA microarray analysis using Affymetrix Gene Arrays. This resulted in the identification of 581 differentially expressed genes, of which 182 were up‐regulated and 399 were down‐regulated following knockdown of *NRP‐1*. Pathway analysis identified 19 canonical pathways that were affected. Importantly, the *Cell Cycle: G2/M DNA Damage Checkpoint Regulation* pathway was the only one with a |z‐score | > 2. IPA also identified 21 significantly changed diseases and bio functions; *proliferation of cells* was decreased and ranked as the highest network with 206 focus molecules. These results demonstrated that *NRP‐1* knockdown may inhibit tumour progression by affecting cell proliferation.

Finally, we measured cellular growth using MTT and colony formation assays. These two measurements indicated that suppression of *NRP‐1* inhibited GBC cell proliferation in vitro, which is consistent with IPA and our in vivo data. Using WB, we then confirmed that suppression of *NRP‐1* increased the expression of BBC3 and EGR1 and decreased the expression of CCNA2 and MDM2, which were all enriched in the IPA *Proliferation of cells* network. Furthermore, we found that *NRP‐1* shRNA blocked the up‐regulation of CCNA2 and MDM2 which mediated by IL‐8, and these results demonstrated that CAFs‐secreted IL‐8 up‐regulated *NRP‐1* in GBC cells and subsequently promoted GBC cell proliferation by decreasing the expression of CCNA2 and MDM2. CCNA2 drives S phase progression by binding to and activating Cdk2 and Cdk1. Then, Cdk/CCNA2 complexes phosphorylate pocket proteins (Rb, p107, p130) and proteins involved in DNA synthesis.^33^ Aberrant expression of CCNA2 has been detected in a variety of cancers, and deregulation of CCNA2 was closely related to tumour proliferation and chromosomal instability.^34^ Inhibition of CCNA2 complexes also has been shown to impair the proliferation of tumour cell lines.^35^, ^36^ MDM2 is an essential regulator of the p53 tumour suppressor, and it is modified at the transcriptional, post‐transcriptional and post‐translational levels to control p53 activity. Higher MDM2 protein expression also results in an increased risk for spontaneous tumour formation.^37^, ^38^, ^39^

Taken together, our study suggests that IL‐8 secretion by CAFs can up‐regulate NRP‐1 in GBC cells and subsequently promote GBC cell proliferation by CCNA2 and MDM2. A new NRP‐1 targeting probe has been be used for the grading diagnosis by MRI of gliomas in nude mice.^40^ As we found that NRP‐1 expression was associated with GBC tumour differentiation, targeting NRP‐1 might also be used for the GBC grading diagnosis. Besides, tumour progression may be retarded by targeting CAFs,^41^ and a tumour‐imaging method targeting CAFs has shown its potential to serve as pantumor agents.^42^ We believe that the CAFs/IL‐8/NRP‐1 axis could be used in developing novel diagnostic and therapeutic strategies for GBC.

## CONFLICTS OF INTEREST

The authors confirm that there are no conflicts of interest.

## AUTHOR CONTRIBUTION

Chen Chen: Conceptualization (equal); Data curation (equal); Formal analysis (equal); Investigation (lead); Methodology (lead); Writing‐original draft (lead). Rui Zhang: Data curation (equal); Investigation (equal); Methodology (equal). Li Ma: Investigation (equal); Methodology (equal). Qi Li: Investigation (equal); Methodology (equal). Yaling Zhao: Methodology (equal); Software (equal); Validation (lead). Guanjun Zhang: Data curation (equal); Methodology (equal). Dong Zhang: Investigation (equal); Methodology (equal). Wenzhi Li: Investigation (equal); Methodology (equal). Sheng Cao: Supervision (equal); Writing‐review & editing (equal). Lin Wang: Conceptualization (equal); Project administration (equal). Zhimin Geng: Conceptualization (lead); Funding acquisition (lead); Writing‐review & editing (lead).

## ETHICS APPROVAL AND CONSENT TO PARTICIPATE

Clinical data have been approved by the Ethics Committee of the First Affiliated Hospital of Xi'an Jiaotong University and approved by the patients. All animal experiments were approved by Animal Care and Use Committee of Xi'an Jiaotong University.

## Supporting information

Table S1‐S3

Data S1

Data S2

Data S3

Data S4

Data S5

## Data Availability

The data that support the findings of this study are available from the corresponding author upon reasonable request.
