# Supplementary material for: Neuropilin‐1 is up‐regulated by cancer‐associated fibroblast‐secreted IL‐8 and associated with cell proliferation of gallbladder cancer
Source: J Cell Mol Med. 2020 Sep 20;24(21):12608–18. doi: 10.1111/jcmm.15825 (PMC7686964; doi:10.1111/jcmm.15825)
Supplement: Supplementary file 1 — Table S1‐S3 [file JCMM-24-12608-s001.doc]

**Supplementary Data Table 1 Top twenty up-regulated genes at NRP-1 KD group**

| Gene symbol | MES at KD | MES at NC | Absolute FC |
| --- | --- | --- | --- |
| ATF3 | 1.0052319 | -0.9333768 | 3.7345965 |
| IL20 | 0.89422846 | -0.80333424 | 3.3150165 |
| SERPINB2 | 0.764987 | -0.7785959 | 2.8546507 |
| KLF4 | 0.70867777 | -0.70331526 | 2.6773038 |
| LRRC49 | 0.647068 | -0.79149437 | 2.6291306 |
| EGR1 | 0.7369814 | -0.62271214 | 2.6046042 |
| DCLK1 | 0.6529422 | -0.6898918 | 2.5841954 |
| PDCD1LG2 | 0.68035793 | -0.6983628 | 2.511303 |
| C3orf52 | 0.65042543 | -0.67882967 | 2.471341 |
| IL24 | 0.68277264 | -0.7494087 | 2.449592 |
| ZNF267 | 0.62648964 | -0.6532607 | 2.3975825 |
| PTGS2 | 0.616025 | -0.6400175 | 2.358461 |
| SESN2 | 0.7020445 | -0.5135236 | 2.2340796 |
| SERPINB7 | 0.5638771 | -0.5463371 | 2.1933239 |
| DUSP5 | 0.52213764 | -0.58298445 | 2.154886 |
| HAS2 | 0.55664825 | -0.5232856 | 2.139882 |
| AADAC | 0.563674 | -0.524467 | 2.1006691 |
| TNFAIP3 | 0.46968746 | -0.6420541 | 2.041579 |
| PTPRK | 0.50716543 | -0.53063345 | 2.0298793 |
| AGT | 0.59400654 | -0.44412613 | 2.0030568 |

MES, mean expression signal; FC, fold change.

**Supplementary Data Table 2 Top twenty down-regulated genes at NRP-1 KD group**

| Gene symbol | MES at KD | MES at NC | Absolute FC |
| --- | --- | --- | --- |
| CNN1 | -1.33325 | 1.2368393 | 5.4971976 |
| PLDN | -1.12549 | 1.0942292 | 4.8238816 |
| LOC100289196 | -1.0025887 | 0.92175484 | 3.969934 |
| LOC100508939 | -1.0025887 | 0.92175484 | 3.969934 |
| AGPS | -0.9599838 | 0.95679283 | 3.8659463 |
| FAM169A | -0.87700033 | 0.8219695 | 3.4490757 |
| ARF3 | -0.8618722 | 0.93011093 | 3.3758168 |
| CD164 | -0.87758875 | 0.83574295 | 3.3167374 |
| KATNAL1 | -0.86596394 | 0.83115864 | 3.2856324 |
| C7orf73 | -0.8357153 | 0.89262056 | 3.208225 |
| SEC23A | -0.82900524 | 0.833024 | 3.156073 |
| ARF6 | -0.7882986 | 0.77704525 | 3.0420425 |
| NRP1 | -0.779953 | 0.9219713 | 3.0363941 |
| RP2 | -0.77318573 | 0.8894892 | 3.034556 |
| RAB40B | -0.92536116 | 0.7336416 | 2.9979057 |
| MBTPS2 | -0.8117237 | 0.8043976 | 2.995514 |
| TMEM33 | -0.6952629 | 0.8272643 | 2.9447284 |
| GLIPR1 | -0.8666129 | 0.70977116 | 2.9318783 |
| AASDHPPT | -0.8301401 | 0.7513361 | 2.9301372 |
| LARP4 | -0.7705283 | 0.76175594 | 2.9184608 |

MES, mean expression signal; FC, fold change.

**Supplementary Data Table 3 List of significantly changed diseases or bio functions.**

| **Diseases or Functions** | **Predicted Activation State** | **z-score** | **Molecules** |
| --- | --- | --- | --- |
| proliferation of cells | Decreased | 4.357 | 206 |
| cell proliferation of tumor cell lines | Decreased | 3.36 | 103 |
| organismal death | Increased | 3.177 | 136 |
| infection of cells | Decreased | 3.151 | 45 |
| morbidity or mortality | Increased | 3.141 | 138 |
| viral infection | Decreased | 3.031 | 91 |
| cytostasis | Decreased | 2.686 | 23 |
| cytostasis of tumor cell lines | Decreased | 2.612 | 13 |
| cell viability of tumor cell lines | Decreased | 2.499 | 58 |
| cell proliferation of colorectal cancer cell lines | Decreased | 2.453 | 22 |
| cell death of connective tissue cells | Decreased | 2.349 | 54 |
| cell death of lung cancer cell lines | Increased | 2.287 | 18 |
| cell death of brain cells | Increased | 2.25 | 18 |
| migration of endothelial cells | Decreased | 2.237 | 25 |
| cell death of melanoma cell lines | Increased | 2.194 | 22 |
| volume of trabecular bone | Increased | 2.189 | 8 |
| cell movement of endothelial cells | Decreased | 2.181 | 27 |
| M phase of tumor cell lines | Decreased | 2.157 | 14 |
| apoptosis of lung cancer cell lines | Increased | 2.08 | 17 |
| cell proliferation of carcinoma cell lines | Decreased | 2.07 | 28 |
| transport of molecule | Decreased | 2.047 | 80 |
| quantity of LDH | Increased | 2 | 4 |
